# Effects of Resistance Exercise with and without Blood Flow Restriction on Acute Hemodynamic Responses: A Systematic Review and Meta-Analysis

**DOI:** 10.3390/life14070826

**Published:** 2024-06-28

**Authors:** Anderson Geremias Macedo, Danilo Alexandre Massini, Tiago André Freire Almeida, Luciana Maria dos Reis, Giovane Galdino, Adriana Teresa Silva Santos, Osvaldo Tadeu da Silva Júnior, Rubens Venditti Júnior, Dalton Muller Pessôa Filho

**Affiliations:** 1 Institute of Motricity Sciences, Federal University of Alfenas (UNIFAL), Alfenas 37133-840, MG, Brazil; andersongmacedo@yahoo.com.br (A.G.M.); luciana.reis@unifal-mg.edu.br (L.M.d.R.); giovane.souza@unifal-mg.edu.br (G.G.); adriana.santos@unifal-mg.edu.br (A.T.S.S.); 2 Pos-Graduation Program in Rehabilitation Sciences, Institute of Motricity Sciences, Federal University of Alfenas, Santa Clara Campus, Alfenas 37133-840, MG, Brazil; 3 Graduate Programe in Human Development and Technology, São Paulo State University (UNESP), Rio Claro 13506-900, SP, Brazil; osvaldo.tadeu@unesp.br (O.T.d.S.J.); r.venditti-junior@unesp.br (R.V.J.); 4 Department of Physical Education, School of Sciences (FC), São Paulo State University (UNESP), Bauru 17033-360, SP, Brazil; dmassini@hotmail.com (D.A.M.); tiagofalmeida.w@gmail.com (T.A.F.A.)

**Keywords:** strength training, cardiovascular responses, kaatsu

## Abstract

Low-load intensity resistance exercise with blood flow restriction (BFR) is an alternative method for enhancing strength and muscle mass. However, acute cardiovascular responses to a complete training session remain uncertain compared to high-load intensity resistance exercise (HI). Therefore, the objective of this study to examine acute and post-exercise hemodynamic responses to low-load BFR and HI protocols. This systematic review and meta-analysis (RD42022308697) followed PRISMA guidelines to investigate whether the responses of heart rate (HR), blood systolic (SBP), blood diastolic pressure (DBP), and rate pressure product (RPP) immediately after and up to 60 min post-exercise from BFR were consistent with those reported after resistance exercises performed at HI in healthy individuals. Searches using PICO descriptors were conducted in databases from January 2011 to December 2023, and effect sizes were determined by Hedge’s *g*. The selected studies involved 160 participants in nine articles, for which the responses immediately after BFR and HI exercises showed no differences in HR (*p* = 0.23) or SBP (*p* = 0.57), but significantly higher DBP (*p* < 0.01) and lower RPP (*p* < 0.01) responses were found when comparing BFR to HI. Furthermore, the BFR and HI protocols showed no differences regarding SBP (*p* = 0.21) or DBP (*p* = 0.68) responses during a 15 to 60 min post-exercise period. Thus, these results indicated that hemodynamic responses are similar between BFR and HI, with a similar hypotensive effect up to 60 min following exercise.

## 1. Introduction

High-load intensity resistance exercise (HI) (≥ 60% of 1RM, one repetition maximum) has been recommended for muscle mass and strength enhancement in healthy subjects [1,2]. However, during HI performance, there is an increase in acute hemodynamic responses, such as heart rate (HR), systolic blood pressure (SBP), diastolic blood pressure (DBP), and cardiovascular overload by rate pressure product (RPP) [3,4]. These hemodynamic adjustments induced by resistance exercise occur due to increased sympathetic nervous activity mediated by central mechanisms and the exercise pressor reflex. Specifically, the exercise pressor reflex involves feedback mechanisms from active muscles known as the mechanoreflex and metaboreflex [5,6]. The mechanoreflex is mediated by afferent fibers of the sympathetic nervous system belonging to Group III, which are sensitive to mechanical stimuli such as muscle tension and deformation during muscle contraction. In contrast, the metaboreflex involves thin afferent fibers of the sympathetic nervous system belonging to Group IV, which respond to biochemical stimuli caused by the accumulation of metabolites due to vessel compression during muscle contraction, such as lactate, inorganic phosphate, and H^+^ ions [6]. Another acute effect of HI is the reduction of SBP and DBP levels after the session, a phenomenon known as post-exercise hypotension (PEH) caused by vasodilator agents that promote endothelial muscle relaxation [7,8]. Although these acute hemodynamic changes occur, experimental clinical results indicate that HI is relatively safe for healthy individuals without significant cardiovascular system overload. However, it is still recommended to monitor cardiovascular responses during its practice, such as the rate pressure product [4,9]. 

In the last decade, low-load intensity resistance exercise (20–30% of 1RM) combined with blood flow restriction (BFR) has also been shown as an alternative method for muscle mass and strength enhancements in healthy individuals (e.g., athletes, young, and older adults) under physical rehabilitation processes or who are unable to lift high-intensity loads [10,11]. BFR also seems to stimulate a sharp increase in hemodynamic responses and hence overloads the cardiovascular system [12,13]. Aside from mechanical tension time stimulus on the mechanoreflex, the increase in cuff pressure leads to partial or total occlusion of blood flow to the limbs or muscle group involved during resistance exercise [14,15], which increases metabolite accumulation, potentiating the metaborreflex, and consequently stimulates exacerbated sympathetic excitation that increases HR, SBP, and DBP [14,15]. In addition, blood flow restriction appears to potentiate PEH due to the greater accumulation of vasodilatory mediators [7,13].

Studies involving healthy subjects performing HI and BFR protocols have supported the increase in hemodynamic responses and cardiovascular overload at the end of the session as well as the PHE post-session occurrence (up to 60 min after the session) [9,16,17,18]. However, the similarities when comparing acute increases in hemodynamic responses, cardiovascular system overload, and antihypertensive effects of the HI and BFR protocols in healthy individuals have yet to be further demonstrated due to the high variance of the responses, most likely caused by different exercises and loads used in the protocols [17,18,19]. Therefore, it is not possible to ensure whether the hemodynamic response trends are similar or different between the protocols. Despite this uncertainty, it seems that BFR training may be relatively safe for healthy individuals, as it has desirable antihypertensive effects and does not cause high overload on the cardiovascular system [7,16,19,20,21].

To date, only three systematic reviews have examined hemodynamic responses between BFR and HI. However, these reviews focused on acute, post-exercise, and chronic responses in healthy individuals and those with pathologies [22,23,24]. In the literature, two meta-analyses investigated the effects of hemodynamic responses involving BFR and HI. Domingos and Polito [23] examined the effect of systolic and diastolic blood pressure during and after resistance exercise in various populations and different blood pressure measurement techniques, while Pedon et al. [24] investigated the effect of blood flow restriction on acute responses of HR, SBP, and DBP in healthy individuals. Although both meta-analyses are important, the acute effects on the rate pressure product and post-exercise responses have not yet been investigated. Therefore, the present study aimed to examine acute hemodynamic responses and post-exercise outcomes through a systematic review and meta-analysis of protocols commonly used in high-load intensity resistance exercise versus low-load intensity resistance exercise with blood flow restriction in healthy individuals.

## 2. Materials and Methods

This systematic review and meta-analysis was carried out following the recommendations of the Cochrane Handbook for Systematic Reviews of Interventions (version 5.1.0) and written according to the Preferred Reporting Items for Systematic Reviews and Meta-Analyses (PRISMA) checklist [25] (see Supplementary File). This systematic review received the registration number CRD42022308697 in the PROSPERO baseline records.

The search for this review was conducted electronically in the National Library of Medicine (PubMed), Web of ScienceTM, SciELO, Science Direct, and Semantic Scholar from January 2011 to December 2023. The search used the Population, Intervention, Comparator, and Outcome (PICO) framework and was conducted using the descriptors “resistance exercise” OR “resistance training” AND “blood flow restriction” OR “kaatsu” AND “blood pressure” OR “hemodynamics”. Only articles published in indexed journals in the English language were included. The search sensitivity was assessed by locating the article by Rossow et al. [16]. Manual searches of the references of eligible articles and their citations in the PubMed, Scopus, and Google Scholar databases were conducted to identify other relevant titles. Gray literature (e.g., abstracts, conference proceedings, editorials, dissertations, and theses) was not included [26]. Attempts were made to email the authors of selected articles to request any missing information. Two authors (AGM and TAFA) performed all the searches to avoid selection bias. After performing the searches, the authors compared the lists of included and excluded studies; the discrepancies observed were analyzed through discussion and agreement with a third author (DMPF).

### 2.1. Study Selection Criteria

The inclusion criteria for this review were adopted as follows: (1) type of studies: peer-reviewed clinical trials and cross-over studies (for studies on acute and post-exercise responses, time periods up to 60 min and trials involving single or sequential exercises were considered); (2) type of subjects: studies performed with healthy adults (between 18 and 70 years) of both sexes, without cardiovascular, metabolic, pulmonary, osteoarticular limitation, and obesity diseases; (3) type of intervention: traditional high-intensity resistance exercise ≥ 60% of 1RM and low-intensity resistance exercise ≤ 30% of 1RM with BFR involving the two protocols in the same session; (4) type of comparison: high-intensity resistance exercise and low-intensity resistance exercise with BFR; (5) outcomes: acute HR (bpm), SBP (mmHg), DBP (mmHg), and RPP (bpm×mmHg) as well as post-exercise SBP (mmHg) and DBP (mmHg).

The exclusion criteria were as follows: (1) studies that used dietary supplements, pharmaceuticals, or ergogenic aids known to alter blood flow or hemodynamic responses; (2) studies involving HI or BFR with subjects with cardiovascular, metabolic, pulmonary, osteoarticular-limiting, and obesity diseases; (3) literature reviews (narrative reviews, systematic reviews, and meta-analyses); and (4) studies with low methodological quality.

### 2.2. Data Extraction

Two authors (AGM and DAM) extracted the data using a pre-pilot spreadsheet and independently verified it with a third author (DMPF) from the review team. The following data were extracted: (i) author names, (ii) year of publication, (iii) population characteristics (sample size, sex, age, height, and body mass), (iv) training method, (v) training load parameters (exercises, volume, intensity, recovery, weekly frequency, and intervention duration), and (vi) pre- and post-training AT.

### 2.3. Methodological Quality Assessment and Risk of Bias

Methodological quality assessment and risk of bias were conducted by two independent authors (LMR and DAM), with discrepancies resolved by a third author (GG) using the 11-point PEDro Scale (Physiotherapy Evidence Database), which assigns 1 point to the study if the criterion is met or 0 if not [27]. As criterion 1 pertains to external validity, it was considered in the total score; likewise, criteria 5, 6, and 7 were removed due to the impossibility in exercise intervention studies of blinding participants’ group allocation as well as the rarity of researchers acting blindly [27]. With the removal of these items, the maximum value of the PEDro scale was 7 points, with adjusted ratings ranging from 0 to 3 indicating “poor quality”, 4 indicating “moderate quality”, 5 indicating “good quality”, and 6 to 7 indicating “excellent quality” [26,28].

Two authors (AGM and DAM) assessed the risk of bias using the second version of the Cochrane risk-of-bias tool for non-randomized studies (ROBINS-I) [29] in the following domains: (i) risk of bias due to confounding; (ii) risk of bias in the selection of participants for the study; (iii) risk of bias in the classification of interventions; (iv) risk of bias due to deviations from intended interventions; (v) risk of bias due to missing data; (vi) risk of bias in the measurement of outcomes; and (vii) risk of bias in the selection of the reported result. A study had a low risk of bias if it was rated as “low risk” in all domains, a moderate risk of bias if there was at least one domain rated as “moderate risk”, a serious risk of bias if there was at least one domain rated as “serious risk” or multiple domains rated as “moderate risk” that may affect the validity of the results, and critical risk of bias if there was at least one domain rated as “critical risk” or multiple domains rated as “serious risk” that may affect the validity of the results. Weighted summary and traffic light risk-of-bias plots for non-randomized included studies were produced by the online risk-of-bias visualization (robvis) tool “URL (acessed on 05 March 2024) [30]. Any discrepancies were resolved through discussion with another author (ATSS).

### 2.4. Statistical Analysis

The statistical analysis was conducted by one author (DAM) and reviewed by a second author (DMPF). For these estimates, the sample size, mean values, and standard deviations of hemodynamic variables (HR, SBP, DBP, and RPP) for each HI and BFR condition in each study included in the meta-analysis were utilized. The magnitude of the outcome for each hemodynamic variable was determined by the standardized mean differences adjusted by Hedge’s *g* and a 95% confidence interval (CI) due to the small sample size (n < 30) of the included studies [31].

The estimates from the studies were combined within the meta-analysis using a random effects model and presented as forest plots. Inconsistency was assessed using the results of the meta-analysis, and it was based on visual inspection of Hedge’s *g* estimates with overlapped or non-overlapped CI_95%_ as well as statistical tests for heterogeneity (*I*^2^) determined by combining Cochran’s Q test with the Higgins test [32]. Its value was classified as follows: 0 < *I*^2^ ≤ 25% indicating no heterogeneity; 25% < *I*^2^ ≤ 50% indicating low heterogeneity; 50% < *I*^2^ ≤ 75% indicating moderate heterogeneity; and *I*^2^ > 75% indicating high heterogeneity among studies [26].

Additional subgroup analyses, sensitivity analyses, and meta-regression were not assessed, nor were publication bias analyses (Egger’s test) due to the limited number (n < 10) of included studies [33,34,35]. The effect size for Hedge’s *g* was categorized as ≤ 0.19 [trivial], 0.20–0.59 [small], 0.60–1.19 [moderate], and ≥ 1.20 [large] [36]. All analyses were conducted using R software (version 4.0.3) and the RStudio environment (version 1.3.1093) with the *meta* [37] and *metafor* [38] packages. A significance level of α = 0.05 was adopted for all statistical procedures.

## 3. Results

**Figure 1:**
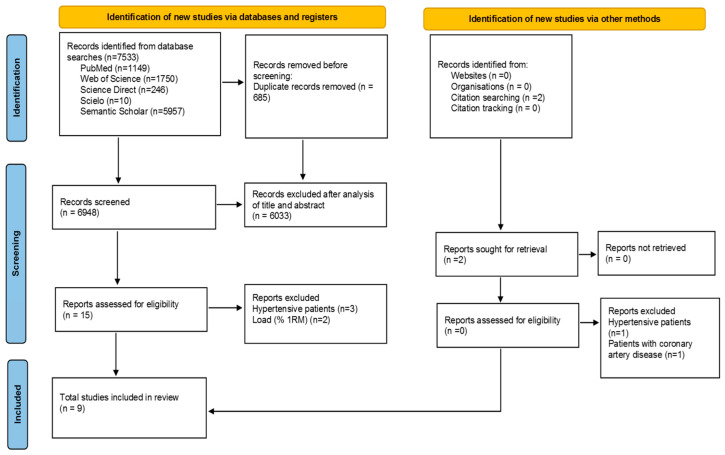
Flow diagram for systematic review.

Figure 1 presents the flowchart of all stages of the systematic review and meta-analysis, while Table 1 outlines the main characteristics of the nine included studies. Five studies [7,17,20,21,39] were conducted in South America, two [18,40] in Oceania, one [16] in the USA, and one [20] in Asia. 

The studies involved 160 participants (79% men and 21% women) between the ages of 18 and 67. Regarding the resistance exercise protocol, one to four exercises were utilized, with those employing one or two exercises focusing on the lower limbs [17,18,39,40], one study using four exercises for lower limbs [16], another for upper limbs [21], and three studies using two exercises for upper limbs and two for lower limbs [7,19,20]. The number of sets per exercise ranged from three to six, with intensity ranging between 20 and 30% 1 RM for BFR and between 70 and 80% 1 RM for HI. The repetitions per set ranged from 10 to 30 for BFR and 8 to 15 for HI. Only one protocol for both BFR and HI was conducted until muscular failure. Finally, the recovery interval between sets was between 0.5 and 1 min for BFR and between 1 and 2 min for HI.

### 3.1. Meta-Analysis

**Figure 2:**
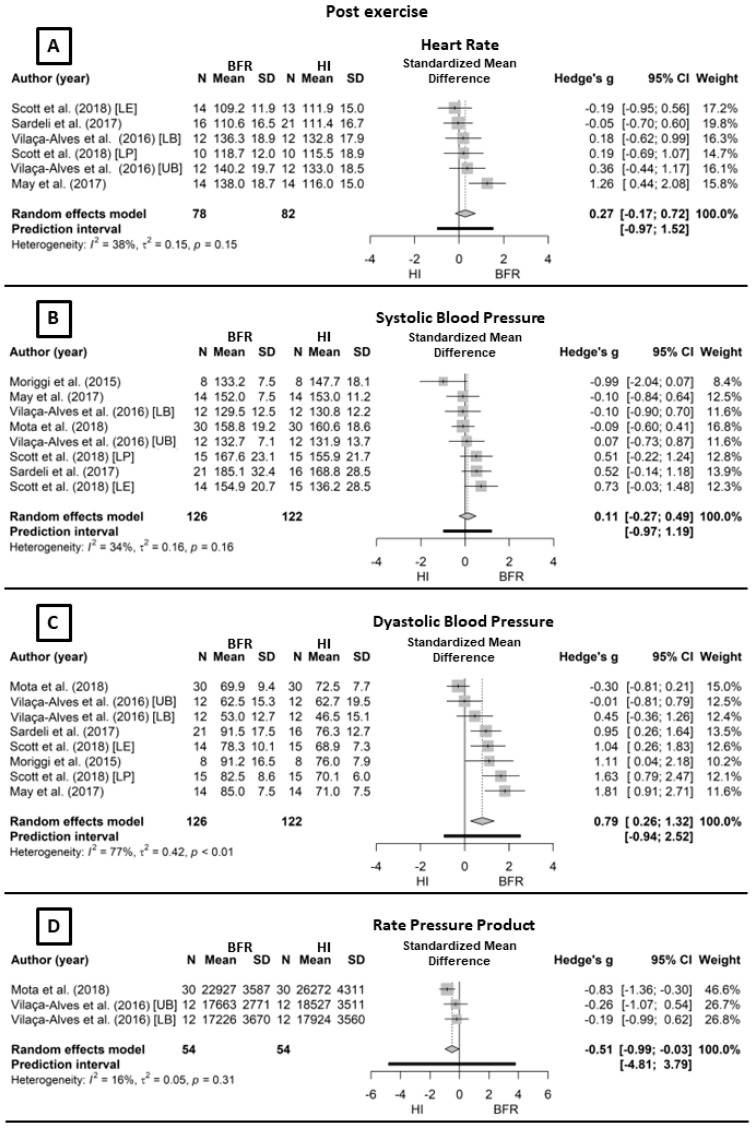
Forest plot of acute responses comparing BFR to HI. Outcomes: (**A**) heart rate, (**B**) systolic blood pressure, (**C**) diastolic blood pressure, (**D**) rate pressure product. CI, confidence interval; BFR, low-intensity resistance exercise with blood flow restriction; HI, high-intensity resistance exercise; SD, standard deviation. Sardeli et al. (2017) refers to reference [17]; Scott et al. (2018) refers to reference [18]; Vilaça-Alves et al. (2016) refers to reference [19]; Moriggi et al. (2015) refers to reference [21]; Mota et al. (2018) refers to reference [39]; and May et al. (2018) refers to reference [40].

Figure 2 depicts the acute hemodynamic responses.

These studies combined by the random-effects model for HR (Panel A) under low heterogeneity (*I*^2^ = 37.9%; Q_[5]_ = 8.05, *p* = 0.15) showed no significant difference between BFR and HI (*g* = 0.27, CI_95%_ = −0.17–0.78, *p* = 0.23, [small]). For systolic blood pressure (Panel B), using the random-effects model under low heterogeneity (*I*^2^ = 33.5%; Q_[7]_ = 10.6, *p* = 0.16), there was also no significant difference between the protocols (*g* = 0.11, CI_95%_ = −0.17–0.49, *p* = 0.57, [trivial]). However, diastolic blood pressure (Panel C), using the random-effects model under high heterogeneity (*I*^2^ = 76.8%; Q_[7]_ = 30.1, *p* < 0.01), showed a significant difference between BFR and HI (*g* = 0.79, CI_95%_ = 0.26–1.32, *p* < 0.01, [moderate]). Finally, the double product (Panel D), using the random-effects model under no heterogeneity (*I*^2^ = 15.7%; Q_[2]_ = 30.1, *p* = 0.30), also showed a significant difference between HI and BFR (*g* = −0.51, CI_95%_ = −0.99–0.03, *p* = 0.04, [small]).

**Figure 3:**
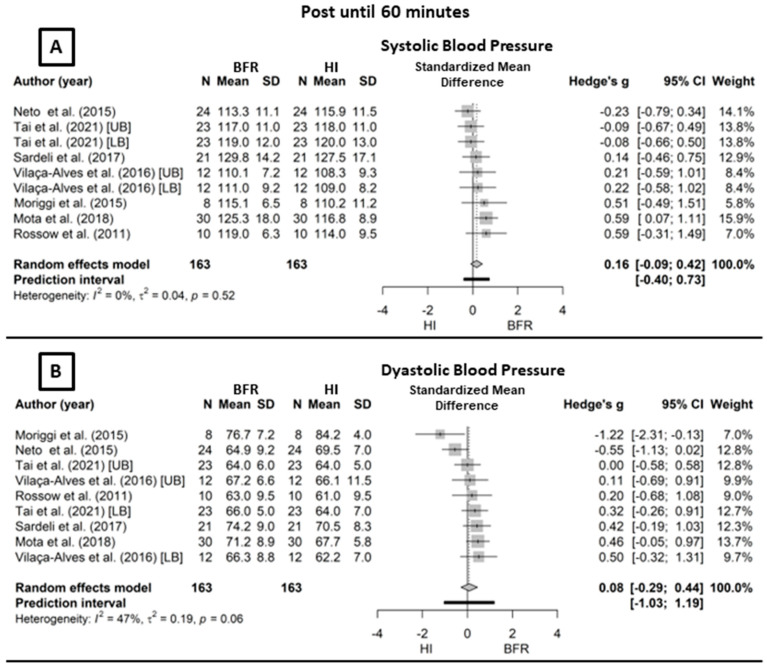
Forest plot of post-exercise responses from 15 to 60 min comparing BFR to HI. Outcomes: (**A**) systolic blood pressure; (**B**) diastolic blood pressure. CI, confidence interval; BFR, low-intensity resistance exercise with blood flow restriction; HI, high-intensity resistance exercise; SD, standard deviation. Neto et al. (2015) refers to reference [7]; Rossow et al. (2011) refers to reference [16]; Sardeli et al. (2017) refers to reference [17]; Vilaça-Alves et al. (2016) refers to reference [19]; Tai et al. (2021) refers to reference [20]; Moriggi et al. (2015) refers to reference [21]; Mota et al. (2018) refers to reference [39].

Figure 3 displays the hemodynamic responses between 15 and 60 min after the exercise. These studies combined by the random-effects model for SBP (Panel A) under no heterogeneity (*I*^2^ = 0.0%; Q_[8]_ = 7.20, *p* = 0.51) showed no significant difference between BFR and HI (*g* = 0.16, CI_95%_ = −0.09–0.42, *p* = 0.21, [trivial]). Similarly, DBP (Panel B) under low heterogeneity (*I*^2^ = 47.0%; Q_[8]_ = 15.1, *p* = 0.06) also did not show a significant difference between the protocols (*g* = 0.08, CI_95%_ = −0.29–0.44, *p* = 0.68, [trivial]).

### 3.2. Methodological Quality and Risk of Bias

Table 2 presents the results of each PEDro methodological quality scale criterion for all included studies. Three studies demonstrated excellent methodological quality [18,39,40], and six were rated good quality [7,16,17,19,20,21]. Thus, the methodological quality score provided by the PEDro scale was 5.3 points (good quality), ranging between 5 and 6 points.

**Figure 4:**
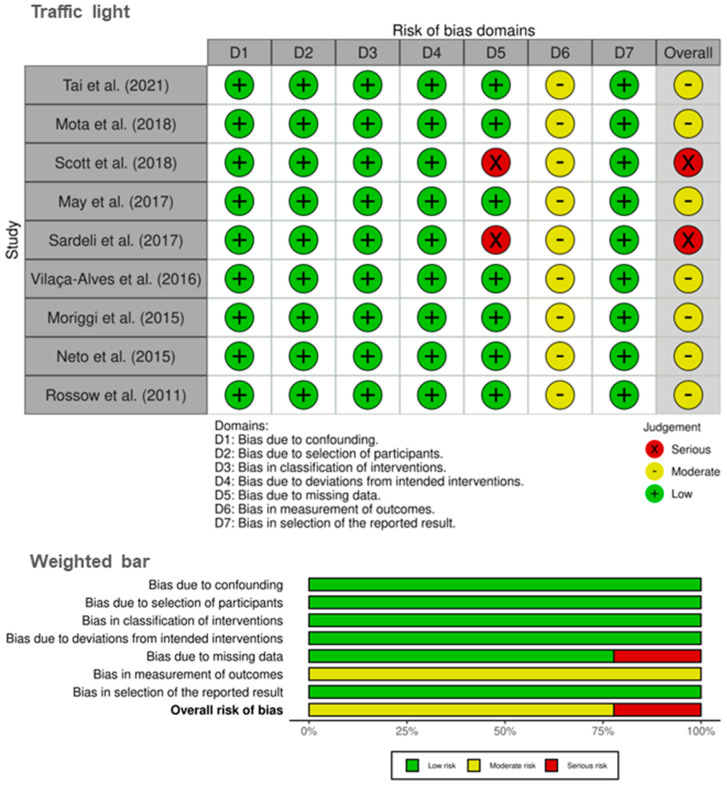
Publication bias analysis. Lower panel: traffic light plot; Upper panel: weighted bar plot. Studies are listed according to the year of publication, from the earliest to the latest published. Tai et al. (2021) refers to reference [20]; Mota et al. (2018) refers to reference [39]; Scott et al. (2018) refers to reference [18]; May et al. (2017) refers to reference [40]; Sardeli et al. (2017) refers to reference [17]; Vilaça-Alves et al. (2016) refers to reference [19]; Moriggi et al. (2015) refers to reference [21]; Neto et al. (2015) refers to reference [7]; Rossow et al. (2011) refers to reference [16].

Regarding the risk of bias presented in the upper panel (traffic light plot) of Figure 4, moderate risks were observed in all studies [7,16,17,19,20,21,39,40] concerning outcome measurement bias. Two studies [17,18] showed a serious risk related to bias due to missing data. The overall risk of bias presented in the lower panel (weighted bar plot) of Figure 4 indicated approximately 78% moderate risk and 22% serious risk.

## 4. Discussion

This study aimed to conduct a systematic literature review and meta-analysis to investigate the hemodynamic responses of HR, SBP, DBP, and RPP immediately post-exercise and the SBP and DBP responses 15 to 60 min post-exercise following BRF and HI protocols. The findings revealed that: (a) post-exercise responses did not demonstrate significant differences in HR and SBP between BFR and HI (however, BFR exhibited higher DBP values and lower RPP compared to HI) and (b) both protocols showed comparable hemodynamic responses from 15 to 60 min post-exercise.

The investigated populations were predominantly composed of male individuals [7,16,19,21,39,40] followed by studies including both sexes [17,20] and only one study of just women [18]. Therefore, the low percentage of women and the lack of separate analyses by sex make it difficult to infer results for women, compare between sexes, and analyze subgroups on the influence of sex and age. Although most studies involved men, an examination of results from studies involving women [18,20] did not show values (mean ± SD) for HR, SBP, and DBP that differed from those in studies involving men. Clearly, a similar magnitude of response was observed between men and women in response to hemodynamic variables during resistance exercise. However, this is a preliminary analysis and still requires theoretical underpinning; at rest, no differences were observed in SBP and DBP [41], but during exercise conditions, there are reports of differences in SBP values between men and women in traditional resistance exercise (without BFR) for the same relative load (%1 RM) and repetitions [42]. Regarding age, it did not appear to influence the findings. It has already been demonstrated that there are no differences in responses to traditional resistance training or BFR [12,43]. However, these two aspects (sex and age) should be explored in future systematic reviews to evaluate potential interferences in hemodynamic responses involving BFR and HI.

The studies analyzed employed different methods for measuring SBP and DBP: five used the oscillometric method [7,16,20,21,40], three used the auscultatory method [18,19,39], and one used the photoplethysmography method [17]. Although different methods of blood pressure measurement were used across the studies, which can yield values susceptible to instrument-specific precision levels, it is important to note that there was no comparison between devices but rather between mean exercise responses. Therefore, factors such as type of exercise, fitness level, and age significantly influence these responses. For instance, the HR, SBP, DBP, and RPP values do not show high dispersion (e.g., SD less than 20%), and their means do not vary more than 2×SD when comparing studies. The studies with the highest variation in mean values compared to others were Vilaça-Alves et al. [19] and Sardeli et al. [17], specifically for HR and SBP, respectively. These variations can be explained by the exercise region in the first and the rest interval in the second study. Thus, the data indicate that the measurement equipment does not influence the results, but the exercise conditions do. Furthermore, the oscillometric and auscultatory methods, which constitute the majority of the selected studies (eight out of nine), are widely recommended in the literature for measuring blood pressure during exercise. These methods show measurement differences ranging from 1.9 to 2.0 mmHg for systolic pressure and 1.5 to 4.0 mmHg for diastolic pressure [44,45]. Such measurement errors are unlikely to significantly affect the observed trend in the results presented in Figure 2 and Figure 3.

The studies investigated had varied positioning and degrees of occlusion or absolute pressure applied in mmHg by the cuff for blood flow restriction; however, these differences did not seem to affect hemodynamic responses. For upper limb placement, the cuff was positioned at the proximal end of the arm [19,21] or the axillary region [7,20]. When positioned on the lower limb, the cuff was placed at the proximal end of the thigh [16,18,20,21] or the inguinal region [7,17,19]. These variations in placement did not affect the results due to their similarity [7,16,17,18,19,20,21,39,40]. Another factor is how blood flow occlusion is achieved by relative blood flow restriction at rest or by applying absolute pressure in mmHg [43]. The studies utilized a 50% blood flow occlusion [17] or absolute pressures ranging from 60 to 220 mmHg [7,19,21,39]. However, this pressure range does not significantly alter blood pressure [46,47]. Therefore, it is suggested that the percentage of occlusion or the pressure applied by the cuff in the investigated protocols did not interfere with the study outcomes.

Typically, the cuff is continuously inflated in resistance exercise protocols with BFR [46,47,48]. The vast majority of investigated training protocols kept the cuff inflated throughout (remained inflated between sets) [7,16,17,18,20,21,39,40], with only one protocol using intermittent inflation (cuff deflated between sets) [19]. Whether the cuff was used continuously or intermittently, no differences were observed in hemodynamic variables after BFR exercise in hypertensive middle-aged and older women [49]. However, further research is needed to understand the relationship between continuous or intermittent BFR and hemodynamic variables after resistance exercise in normotensive individuals.

Regarding cuff sizes, the literature suggests that wider cuffs (>13.5 cm) are more effective for blood flow restriction as they require lower applied pressure compared to narrower cuffs (<13.5 cm) [24,50,51]. Among the included studies, three used narrow cuffs for upper limbs [7,19,20], while for lower limbs, two used wide [17,21] and six used narrow cuffs [7,16,19,20,39,40]. In studies on both upper and lower limbs, using wide [17] or narrow cuffs [19] resulted in similar hemodynamic responses at the end of the exercise session or 60 min afterward. Although its interference with hemodynamic parameters has not yet been investigated, cuff size has been shown to alter arterial diameter or blood flow velocity, factors that directly influence blood pressure [52].

The hemodynamic responses observed in BFR and HI protocols of the studies analyzed occurred after the execution of either a single isolated exercise [16,17,18,39,40] or multiple exercises performed either together [19] or in different body segments [7]. Regarding the performance of a single exercise for the lower limbs, multi-joint exercises showed higher hemodynamic values than single-joint exercises in both BFR and HI protocols [18]. Although this comparison was not observed for the upper limbs, it is plausible that the results would be similar due to the greater muscular recruitment and release of metabolites promoted by multi-joint exercises, which directly affect hemodynamic responses [3,5]. Regarding the sequence of exercises in protocols with only single-joint exercises [19,20] or multi-joint and single-joint exercises [20], the authors did not analyze them. However, regardless of the sequential order, the magnitude of hemodynamic responses after exercise was not altered in normotensive middle-aged and older individuals [52]. Regarding the comparison of hemodynamic responses between upper and lower body exercises, Vilaça-Alves et al. [19] found no differences in hemodynamic values at the end or 60 min after exercises, which was supported by Tai et al. [20], who found no differences in hemodynamic variables 55 min after upper and lower body exercises.

The hemodynamic responses in the studies analyzed across BFR and HI protocols were observed after the execution of either a single isolated exercise [16,17,18,39,40] or multiple exercises, either performed together [19] or in different body segments [7]. Regarding the performance of a single exercise for the lower limbs, multi-joint exercises showed higher hemodynamic values than single-joint exercises in both BFR and HI protocols [18]. Although this comparison was not observed for the upper limbs, it is plausible that the results would be similar due to the greater muscular recruitment and release of metabolites promoted by multi-joint exercises, which directly affect hemodynamic responses [3,5]. Regarding the sequence of exercises in protocols with only single-joint exercises [19,20] or multi-joint and single-joint exercises [20], the authors did not analyze them. However, regardless of the sequential order, the magnitude of hemodynamic responses after exercise was not altered in normotensive middle-aged and older individuals [53]. Regarding the comparison of hemodynamic responses between upper and lower body exercises, Vilaça-Alves et al. [19] found no differences in hemodynamic values at the end or 60 min after exercises, which was supported by Tai et al. [20], who found no differences in hemodynamic variables 55 min after upper and lower body exercises.

Some variables in resistance training seemed to influence hemodynamic responses in the studies investigated, primarily the load (%1 RM) and the number of sets. In BFR protocols, loads of 30% [17] or 20% of 1 RM were utilized [7,16,18,19,20,21,39,40], while in HI, loads of 80% [7,17,40] or 70% of 1 RM were used [16,18,19,20,21,39]. The results showed that BFR intensities of 20% to 30% of 1 RM or 70% to 80% of 1 RM produced similar HR and SBP responses at the end, as well as SBP and DBP up to 60 min after exercise. The higher DBP response and lower RPP in BFR after exercise may be due to the higher peripheral vascular resistance and lower cardiac output found; however, there is no statistical support for this within the analyzed studies [17,18,21]. Regarding the number of sets for BFR and HI, three to four sets were utilized [16,17,18,19,20,21,40], with one study using six sets [39]. However, considering the total number of sets across protocols, it ranged from 3 to 16, with the highest number of sets resulting in a greater reduction in DBP, albeit without statistical support, in the BFR protocol 60 min after exercise [7].

A primary challenge in conducting meta-analyses and establishing robust evidence regarding the effects of blood flow restriction exercise is the heterogeneity present in the methodologies of the studies. However, the heterogeneity in this meta-analysis pertains to differences in intervention protocols (e.g., number of exercises, sets, body segments, occlusion site, and cuff size) [54]. Therefore, employing a random-effects model during meta-analysis is advantageous, as it weighs studies relatively more equitably than fixed-effects models [55].

Therefore, the small effect size post-exercise for HR and the trivial effect for SBP between the protocols demonstrate that both present relative cardiovascular safety, as HI is a clinically safe method from a cardiovascular standpoint [4]. Regarding the moderate effect of DBP and small effect of RPP between the protocols, these do not signify significant cardiovascular overload, indicating that BFR can be prescribed [4,23]. At 60 min post-exercise, the trivial effect of SBP and DBP between the protocols demonstrates that both promoted a hypotensive effect [7,23].

Regarding the methodological quality (PEDro = 5.3) of this systematic review [27], its results stemmed from studies with good methodological quality [28]. However, in some cases, its score revealed limitations in outcome measurements. Nevertheless, this is essential for interpreting the study results [56]. Thus, the results were juxtaposed with the risk of bias [29], and it was observed that in all included studies, both investigators and participants were aware of the BFR and HI protocols, which could introduce bias in the outcomes. The studies by Sardeli et al. [17] and Scott et al. [18] exhibited bias due to missing data.

Based on the analyzed data, BFR practice from a cardiovascular safety perspective can involve both multi-joint and single-joint exercises for upper and lower limbs, comprising three to six sets at 20–30% of 1 RM, with continuous absolute pressures ranging from 60 to 160 mmHg between sets. In addition to BFR, other training modalities, such as functional and calisthenics training, appear to demonstrate cardiovascular safety during their practice among healthy individuals [57,58]. BFR can also be used for individuals affected by various conditions such as advanced age, cardiovascular diseases, hypertension, diabetes, orthopedic limitations, and renal dysfunction. However, when planning BFR exercises for individuals with such clinical conditions, it is advisable to consider the augmented acute hemodynamic responses to exercise compared to healthy individuals [23]. Moreover, the rising metabolites in response to the BFR maneuver might, per se, stimulate these hemodynamic responses to increase further [14,23]. Considering these demands on cardiovascular response and the tendency of thrombosis and blood clot formation in clinical individuals [15], the risk of cardiovascular disturbance while exercising with BRF should be addressed in future investigations before making unrestrictive recommendations for therapeutic purposes.

### Study Limitations

The limitations found in the studies that affected the present systematic review and meta-analysis are as follows: (a) a low number of studies, which limited the sample size to only healthy individuals; (b) differences in methods for measuring blood pressure; (c) the low number of participants, which did not allow for the investigation of hemodynamic responses in relation to subgroups based on sex or age among healthy individuals; (d) wide variations in exercises, both single-joint and multi-joint, as well as for different body segments, making subgroup analyses related to the type of exercise and body segment challenging. Therefore, future studies should include (i) separate analyses for women as well as comparison between sexes and across different age groups; (ii) examination of cuff size and positioning; (iii) investigation of the isolated and combined effects of single-joint and multi-joint exercises on both immediate and 60-min hemodynamic responses in both the same and different body segments; and (iv) assessment of the potential long-term effects of BFR (20–30% 1 RM) and HI (70–80% 1 RM) sessions on heart rate, blood pressure, strength gains, and muscle mass across different populations.

## 5. Conclusions

The reviewed studies demonstrated that BFR and HI yielded similar values for HR and SBP post-exercise; however, BFR exhibited higher values for DBP, while HI showed higher values for RPP post-exercise. Nevertheless, both protocols showed no difference in SBP and DBP between 15 and 60 min post-exercise. Overall, BFR is a method that offers similar cardiovascular safety to HI at the end of exercise execution, and both protocols induce a hypotensive effect in the period between 15 to 60 min after resistance exercise. However, these findings might not be extended to subjects with cardiovascular, metabolic, pulmonary, osteoarticular-limiting, and obesity diseases since their compromised physiological responses might alter their hemodynamics compared to healthy individuals.

## Figures and Tables

**Table 1:** Summary of the studies’ results for the acute effects and post-exercise of high-intensity and low-intensity resistance exercise with BFR on heart rate, systolic blood pressure, diastolic blood pressure, and rate pressure product.

Author	Sample	Exercise Protocol	Training Protocol	Hemodynamic Variables and Measurement Moments	Acute Responses	Post-Exercise Response
Neto et al. [7]	24 recreationally trained men (21.7 ± 3.2 years), not informed	Biceps curl Triceps extension Knee extension Knee flexion	BFR: 20%-1RM with 80% pressure, complete occlusion in resting conditions. (4 sets × 30, 15, 15 rep) 30 min rest between all sets and 1 min between exercises. HI: 80%-1RM (4 sets × 8 rep) 2 min rest between all sets and 1 min between exercises.	SBP and DBP; Pre-exercise; Post-exercise 60 min.	Not measured	Post-exercise 60 min HI: SBP (↓4.7 mmHg) DBP (↓2.6 mmHg) BFR: SBP (↓6.6 mmHg) DBP (0.0 mmHg)
Rossowet al. [16]	10 recreationally active men (28.0 ± 5.0 years) (77.3 ± 11.2 kg) (1.76 ± 0.07 m)	Leg press Knee flexion Knee extension Flexion plantar	BFR: 20%-1RM with cuff inflated at a pressure of 250 mmHg (4 sets × 30, 15, 15, 15 rep), 30 s rest between all sets and all exercises. HI: 70%-1RM (3 sets × 10 rep), 1 min rest between all sets and all exercises.	HR, SBP, and DBP; Pre-exercise; Post-exercise 60 min.	Not measured	Post-exercise 60 min HI: HR (↑18.0 bpm) SBP (↓7.0 mmHg) DBP (↓3.0 mmHg) BFR: HR (↑5.0 bpm) SBP (↑1.0 mmHg) DBP (0.0 mmHg)
Sardeli et al. [17]	24 healthy older adults, 12 men and 9 women (64.3 ± 5.0 years) (67 ± 12 kg) (1.63 ±0.08 m)	Leg press	BFR: 30%-1RM with 50% blood flow occlusion (4 sets × 30, 15, 15, 15 rep), 1 min rest between all sets. HI: 80%-1RM (4 sets × voluntary failure, with mean repetitions 9.6 for set), 1 min rest between all sets.	HR, SBP, and DBP; Pre-exercise; Immediately after session; Post-exercise 30 min.	Immediately after session HI: HR (↑44.7 bpm) SBP (↑40.4 mmHg) DBP (↑8.3 mmHg) BFR: HR (↑44.1 bpm) SBP (↑56.0 mmHg) DBP (↑20.6 mmHg)	Post-exercise 30 min HI: HR (↑9.7 bpm) SBP (↓1.0 mmHg) DBP (↑2.5 mmHg) BFR: HR (↑10.3 bpm) SBP (↑0.8 mmHg) DBP (↑3.2 mmHg)
Scott et al. [18]	15 older women (66.8 ± 3.8 years) (65 ± 14 kg) (1.64 ± 0.06 m)	Leg press Leg extension	BFR: 20%-1RM with 50% pressure arterial systolic occlusion (3 sets × 20, 15, 15 rep), 1 min rest between all sets and 8 min between all exercises. HI: 70%-1RM (3 sets × 10 rep), 30 s rest between all sets and 8 min between all exercises.	HR, SBP, and DBP; Pre-exercise; 1st set; 2nd set; 3rd set.	Immediately after set Leg press HI: SBP (↑36.1 mmHg); DBP (↑1.2 mmHg) BFR: SBP (↑47.4 mmHg); DBP (↑11.9 mmHg) Leg extension HI: SBP (↑16.9 mmHg); DBP (↓0.2 mmHg) BFR: SBP (↑40.4 mmHg); DBP (↑7.3 mmHg)	Not measured.
Vilaça-Alves et al. [19]	12 recreationally trained men (22.9 ± 1.9 years) (72.4 ± 4.6 kg) (1.75 ± 0.46 m)	Arm curl Arm extension Knee extension Knee flexion	BFR: 20%-1RM with 180 mmHg on the upper and 220 mmHg on the lower limbs’ occlusion by cuff (3 sets × 15 rep), 30 min rest between all sets and 1 min between exercises. HI: 80%-1RM (3 sets × 10 rep), 90 s rest between all sets and 60 s between exercises.	HR, SBP, DBP, and DP; Pre-exercise; Immediately post-exercise; Post-exercise 60 min.	Immediately after session Upper body HI: HR (↑69.5 bpm); SBP (↑22.2 mmHg) DBP (↓1.9 mmHg); RPP (↑19,755 bpm × mmHg) BFR: HR (↑23.3 bpm); SBP (↑0.6 mmHg) DBP (↓7.6 mmHg); RPP (↑9933 bpm × mmHg) Lower body HI: HR (↑63.1 bpm); SBP (↑20.2 mmHg) DBP (↓14.1 mmHg); RPP (↑9845 bpm × mmHg) BFR: HR (↑56.7 bpm); SBP (↑18.7 mmHg) DBP (↓7.6 mmHg); RPP (↑8793 bpm × mmHg)	Post-exercise 60 min Upper body HI: HR (↓6.7 bpm) SBP (↓1.1 mmHg) DBP (↓1.4 mmHg) BFR: HR (↓6.7 bpm) SBP (↓1.1 mmHg) DBP (↓1.4 mmHg)
Tai et al. et. [20]	23 healthy individuals (14 men and 9 women) (22 ± 2 years) (72.3 ± 12.1 kg) (1.72 ± 0.09 m)	(Upper body) Pulldown Chest press (Lower body) Leg extension Leg curl	BFR: 20%-1RM with 40% blood flow occlusion (4 sets × 30, 15, 15,15 rep), 30 s rest between all sets and 2 min between all exercises. HI: 70%-1RM (4 sets × 8 rep), 1 min rest between all sets and 2 min between all exercises.	HR, SBP, and DBP; Pre-exercise; Post-exercise 55 min.	Not measured	Post-exercise 55 min (Upper Body) HI: HR (↑6.0 bpm) SBP (↑1.0 mmHg) DBP (↓1.0 mmHg) BFR: HR (↑5 bpm) SBP (0.0 mmHg) DBP (↓1.0 mmHg) (Lower Body) HI: HR (↑6 bpm) SBP (↑3.0 mmHg) DBP (0.0 mmHg) BFR: HR (↑4 bpm) SBP (↑2.0 mmHg) DBP (↑1.0 mmHg)
Moriggi et al. [21]	8 young males (23.8 ± 4 years) (74 ± 3 kg) (174 ± 4 cm)	Biceps with dumbbells Biceps scott Closed bench press Triceps extension	BFR: 20%-1RM with cuff occluding 50% of the total occlusion pressure (3 sets × 15, 15, 15 rep), 1 min rest between all sets and all exercises. HI: 70%-1RM (3 sets × 10 rep), 1 min rest between all sets and all exercises.	SBP and DBP; Pre-exercise; Immediately post-exercise; Post-exercise 60 min.	Immediately after session HI: SBP (↑28.0 mmHg) DBP (↓3.0 mmHg) BFR: SBP (↑12.0 mmHg) DBP (↑1.0 mmHg)	Post-exercise 60 min HI: SBP (↓8.0 mmHg) DBP (↑5.0 mmHg) BFR: SBP (↓6.0 mmHg) DBP (↑0.2 mmHg))
Mota et al. [39]	30 healthy active men (27.2 ± 6.8 years) (79 ± 10 kg) (1.78 ± 0.75 m)	Squat	BFR: 20%-1RM with cuff occlusion of 140 mmHg–160 mmHg (6 sets × 10–15 rep), 90 s rest between all sets. HI: 70%-1RM (6 sets × 10–15 rep), 90 s rest between all sets.	SBP, DBP, and DP; Pre-exercise; Immediately after session; Post-exercise 15 min.	Immediately after session HI: SBP (↑30.7 mmHg) DBP (↑2.7 mmHg) SPP (↑17,019 bpm × mmHg) BFR: SBP (↑34.3 mmHg) DBP (↓4.9 mmHg) RPP (↑13,933 bpm × mmHg)	Post-exercise 15 min HI: SBP (↓6.1 mmHg) DBP (0.0 mmHg) BFR: SBP (↑0.8 mmHg) DBP (↑6.2 mmHg)
May et al. [40]	14 recreational young men (22 ± 1 years) (74 ± 9 kg) (1.79 ± 0.06 m)	Leg press	BFR: 20%-1RM with 100 mmHg occlusion (4 sets × 30, 15, 15 rep), 30 s rest between all sets. HI: 80%-1RM (4 sets × 8 rep), 1 min rest between all sets.	HR, SBP, and DBP; Pre-exercise; Immediately after session.	Immediately after session HI: HR (↑62.0 bpm) SBP (↑26.0 mmHg) DBP (↑3.0 mmHg) BFR: HR (↑45 bpm) SBP (↑25.0 mmHg) DBP (↑17.0 mmHg)	Not measured

Abbreviations: kg, kilograms; m, meter; BFR, low-intensity resistance exercise with blood flow restriction; HI, high-intensity resistance exercise; HR, heart rate; SBP, systolic blood pressure; DBP, diastolic blood pressure; RPP, rate pressure product; ↑, increase compared to pre-exercise; ↓, reduction compared to pre-exercise.

**Table 2:** Methodological quality assessment using the PEDro scale.

Studies	Criterion								Scores	Adjusted Ratings
	1	2	3	4	8	9	10	11		
Neto et al. [7]	1	0	0	1	1	1	1	1	5	Good quality
Rossow et al. [16]	1	0	0	1	1	1	1	1	5	Good quality
Sardeli et al. [17]	1	0	0	1	1	1	1	1	5	Good quality
Scott et al. [18]	1	1	0	1	1	1	1	1	6	Excellent quality
Vilaça-Alves et al. [19]	1	0	0	1	1	1	1	1	5	Good quality
Tai et al. [20]	1	0	0	1	1	1	1	1	5	Good quality
Moriggi et al. [21]	0	0	0	1	1	1	1	1	5	Good quality
Mota et al. [39]	1	1	0	1	1	1	1	1	6	Excellent quality
May et al. [40]	1	1	0	1	1	1	1	1	6	Excellent quality

## Data Availability

Not applicable.

## Supplementary Materials

The following supporting information can be downloaded at: https://www.mdpi.com/article/10.3390/life14070826/s1, Figure S1: PRISMA Checklist.
